# Postoperative Acute Kidney Injury After Intraoperative Hypotension in Major Risk Procedures

**DOI:** 10.7759/cureus.64579

**Published:** 2024-07-15

**Authors:** Patrícia Martins Lima, Luana Ferreira, Ana Lídia Dias, Diana Rodrigues, Fernando Abelha, Joana Mourão

**Affiliations:** 1 Department of Anesthesiology, Unidade Local de Saúde São João, Porto, PRT; 2 Center for Research in Health Technologies and Health Systems (CINTESIS), Faculty of Medicine of Porto, Porto, PRT; 3 Physiology and Surgery, Faculdade de Medicina da Universidade do Porto, Porto, PRT

**Keywords:** major surgery, non-cardiac surgery, postoperative complications, hypotension, intraoperative complications, acute kidney injury

## Abstract

Background

Reportedly prevalent, intraoperative hypotension (IOH) is linked to kidney injury and increased risk of mortality. In this study, we aimed to assess IOH incidence in high-risk non-cardiac surgery and its correlation with postoperative acute kidney injury (PO-AKI) and 30-day postoperative mortality.

Methodology

This retrospective cohort study included adult inpatients who underwent elective, non-cardiac, high-risk European Society of Anaesthesiology/European Society of Cardiology surgery from October to November of 2020, 2021, and 2022, excluding cardiac, intracranial, or emergency surgery. IOH was primarily defined by the 2022 Anesthesia Quality Institute. PO-AKI was defined as an increase in serum creatinine ≥0.3 mg/dL within 48 hours, the need for dialysis in dialysis-naïve patients, or the documentation of AKI in clinical records. For univariate analysis, the Mann-Whitney U test and chi-square or Fisher’s exact tests were performed, as appropriate. Logistic regression was used to test risk factors for IOH in univariate analysis (p < 0.1). The significance level considered in multivariate analysis was 5%.

Results

Of the 197 patients included, 111 (56.3%) experienced IOH. After adjustment, surgical time >120 minutes remained associated with higher odds of IOH (odds ratio (OR) = 9.62, 95% confidence interval (CI) = 2.49-37.13), as well as combined general + locoregional (vs. general OR = 3.41, 95 CI% = 1.38-8.43, p = 0.008; vs. locoregional OR = 6.37, 95% CI = 1.48-27.47). No association was found between IOH and 30-day postoperative mortality (p = 0.565) or PO-AKI (p = 0.09). The incidence of PO-AKI was 14.9% (27 patients), being significantly associated with higher 30-day postoperative mortality (p = 0.018).

Conclusions

Our study highlights the high prevalence of IOH in high-risk non-cardiac surgical procedures. Its impact on PO-AKI and 30-day postoperative mortality appears less pronounced compared to the significant implications of PO-AKI, emphasizing the need for PO-AKI screening and renal protection strategies.

## Introduction

The vasodilatory and cardiodepressive effects of anesthesia, as well as absolute hypovolemia during surgical procedures, are frequently major causes of intraoperative hypotension (IOH) [1]. During surgery, most patients have at least one episode of hypotension, with reported incidences differing based on its definition [2,3]. Older patients and those undergoing major surgery are most vulnerable to IOH [4].

A growing body of evidence has revealed a potential connection between the occurrence of IOH and postoperative complications regarding both morbidity and mortality [5]. Extensive observational studies have established a significant association between IOH and postoperative acute kidney injury (PO-AKI), with the risk of organ impairment corresponding with the severity and length of the hypotensive episodes [6]. The likelihood of developing AKI after surgery is mostly determined by baseline variables such as age and cardiovascular condition. Even so, intraoperative low blood pressure has been linked to AKI and mortality. Unlike other risk factors, intraoperative hypotension can be potentially modified [7].

Nonetheless, studies reveal that the incidence of AKI, as defined by the Kidney Disease Improving Global Outcome (KDIGO) criteria, is approximately 6%. Moreover, even mild forms of AKI in non-cardiac surgery patients have been shown to significantly increase the risk of mortality and prolonged hospitalization stay [8,9].

In this study, we sought to investigate the relationship between IOH, PO-AKI, and 30-day postoperative mortality in patients undergoing elective, high-risk, non-cardiac surgery.

## Materials and methods

Study design and setting

We performed a single-center retrospective study including adult inpatients submitted to elective non-cardiac surgery from a Portuguese academic central hospital, based in Porto, Unidade Local de Saúde de São João. We selected patients who underwent the European Society of Anaesthesiology (ESA)/European Society of Cardiology (ESC) high-risk surgery in October and November of 2020, 2021, and 2022.

Participants

Inclusion criteria were defined as adult patients proposed for high-risk surgery (according to ESC/ESA guidelines on non-cardiac surgery) [10] under general, regional, or combined anesthesia. We excluded patients proposed for low and intermediate-risk surgery, cardiac or intracranial surgery, and urgent/emergent surgery.

Data regarding the preoperative period was gathered from electronic medical records, including age, gender, surgical risk (according to ESC/ESA guidelines on non-cardiac surgery) [10], American Society of Anesthesiologists Physical Status (ASA) [11], comorbidities (diabetes, chronic kidney disease (CKD), cardiovascular disease, pulmonary disease, diabetes), and anti-hypertensive therapy.

Intraoperative period data was obtained from surgeries’ electronic anesthesia reports and included anesthetic technique performed (general anesthesia, combined general + regional, regional, sedation), blood pressure monitoring data, surgical specialty, and procedure length. Administration of transitory hypertensive drugs (intravenous boluses of ephedrine and/or phenylephrine) was also considered.

Intraoperative hypotension

Intraoperative blood pressure data were obtained at intervals of up to five minutes for non-invasive blood pressure monitoring and every minute for invasive blood pressure monitoring. Either an automated non-invasive cuff was used to take blood pressure readings every three to five minutes or a continuous indwelling arterial line was used, giving preference to the arterial line data when both methods were used.

The following events were considered artifacts and were removed: mean arterial pressure (MAP) ≥300 or ≤20 mmHg, abrupt changes in MAP defined by greater than or equal to 80 mmHg within five minutes in either direction.

IOH was primarily defined based on the quality indicator from the 2022 Anesthesia Quality Institute (measure ID IIM025): a 15-minute cumulative period of MAP ≤65 mmHg [12]. The total number of minutes of MAP ≤65 mmHg, the maximum total consecutive number of minutes of MAP ≤65 mmHg, and the number of 15-minute consecutive periods of MAP ≤65 mmHg were also considered. The percentage of procedure time with MAP ≤65 mmHg was also computed.

Postoperative acute kidney injury

PO-AKI was defined following the KDIGO criteria and considered present when the following conditions were satisfied: an increase in serum creatinine ≥0.3 mg/dL within 48 hours or the need for dialysis in a patient not previously undergoing dialysis [13]. Regarding the postoperative period, medical electronic records of all study participants were reviewed to evaluate the occurrence of PO-AKI, also included in the study’s PO-AKI defining criteria. Patients with CKD undergoing dialysis and patients with a history of AKI in the previous 30 days were excluded from the analysis.

Statistical analysis

Continuous variables were described as median and interquartile range (IQR), and absolute and relative frequencies were reported for categorical variables. For relevant proportions, 95% confidence intervals (95% CIs) were also reported. For univariate analysis of independent variables and the outcomes of postoperative AKI and mortality, the Mann-Whitney U test was used for continuous variables and the chi-square or Fisher’s exact test for categorical variables, as appropriate. The relationship between MAP and other predictors was also evaluated. The significance level was considered at 5%. The median difference and respective 95% CI were estimated using the Hodges-Lehman estimator.

Multivariable logistic regression was used to assess the association between IOH main definition and postoperative AKI and mortality. All potentially confounding variables (with univariate relationship with postoperative AKI or mortality, p < 0.1, and age) were forced into the models. Bonferroni correction was used to adjust for the independent variables in the models. The Hosmer-Lemeshow p-values were reported to assess the model fit. All analyses were performed using SPSS Statistics Version 28 (IBM Corp., Armonk, NY, USA) [14] and R Core Team (2023) [15].

## Results

Of the 197 patients included in the study, the median age (IQR) was 63 (55-70) years, and 35.5% of the patients were female (Table 1). All patients had preoperative anti-hypertensive therapy in their regular medication history (either angiotensin-converting enzyme inhibitors/angiotensin receptor antagonists, beta-blockers, or diuretics). In total, 45 patients had diabetes, with 55.6% (25 patients) needing insulin therapy for diabetes control in the previous three months. Overall, 20 (10.2%) patients had a history of previous CKD, with 50.0% (nine patients under hemodialysis and one under peritoneal dialysis) under dialysis. Six patients (3.0%) had a history of acute kidney disease in the previous 30 days, with one patient needing renal replacement therapy in this context.

**Table 1:** Demographic characteristics of participants. ACE = angiotensin-converting enzyme; ARA = angiotensin receptor antagonist; AKI = acute kidney injury; ASA = American Society of Anesthesiologists; BNP = brain natriuretic peptide; IQR = interquartile range; Hs-TnI = high-sensitivity troponin I; MAC = monitored anesthesia care

Characteristics	Participants (N = 197)
Age, median, (IQR), years	63 (55–70)
Female	70 (35.5)
ASA class	
I	3 (1.7)
II	62 (35.4)
III	93 (53.1)
IV	17 (9.7)
Missing	22 (11.2)
Surgical specialty	
General surgery	102 (51.8)
Orthopedics	1 (0.5)
Urology	24 (12.2)
Vascular surgery	70 (35.5)
Previous medical history	
Cardiovascular disease	112 (56.8)
Previous myocardial infarct	17 (8.6)
Chronic renal disease	20 (10.2)
Under dialysis	10 (5.1)
AKI in the previous 30 days	6 (3.0)
AKI in the previous 30 days with a need for dialysis	1 (0.5)
Pulmonary disease	40 (20.3)
Diabetes	45 (22.8)
Insulin therapy	25 (12.7)
Cardiac medication history	197 (100)
ACE/ARA inhibitor	84 (42.6)
Beta-blocker	25 (12.7)
Diuretic	24 (12.2)
Preoperative, median (IQR)	
Hs-TnI, ng/L	7.4 (2.7–20.3)
Missing	142 (71.6)
BNP, pg/mL	66 (20–218)
Missing	152 (77.2)
Creatinine, mg/dL	0.77 (0.63–0.99)
Missing	0
Intraoperative	
Surgical time, median (IQR), minutes	289 (179–442)
Missing	11 (5.5)
Anesthesia technique	
General anesthesia	81 (41.1)
Locoregional	38 (19.3)
Combined	72 (36.5)
MAC/Sedation	5 (2.5)
Missing	1 (0.5)

Intraoperative hypotension

The incidence of IOH, according to the definition of a 15-minute cumulative period of MAP ≤65 mmHg, was 56.3% (111 patients) (95% CI = 49.4-63.1%). The median (IQR) cumulative time of IOH was 22 (0-71 minutes), and the median (IQR) maximum total consecutive number of minutes of MAP ≤65 mmHg was zero (0-29) minutes for all patients vs. 26 (15-42) minutes for patients with IOH. Patients with IOH were exposed to a median of one (1-2) 15-minute consecutive periods of MAP ≤65 mmHg, representing 16% (8-30%) of procedure time. When considering only a 15-minute consecutive period of MAP ≤65 mmHg, the incidence of IOH was 44.7% (88 patients) (95% CI = 37.8-51.6%) (comparison with our main definition, considering cumulative time, chi-square test, p = 0.027).

Overall, intermittent boluses of vasopressors (ephedrine and/or phenylephrine) were administered in 80 (72.1%) patients with IOH and 10 (11.6%) patients without IOH.

IOH was significantly related to longer procedure time (Mann-Whitney U test, p < 0.001), specialty (lower incidence for vascular surgery (22.9%) vs. orthopedics (100%), general surgery (71.6%), and urology (87.5%), chi-square test, p < 0.001), smaller ASA (incidence of IOH in ASA <3 of 70.8% vs. 46.4% for ASA ≥3, chi-square test p = 0.003), and anesthesia technique (incidence of IOH in combined general and locoregional anesthesia was 83.3% (44 out of 81), 54.3% for general anesthesia (60 out of 72), 20% for sedation (one out of five), and 15.8% for locorregional anesthesia (six out of 38), Fisher’s exact test p < 0.001), but not with gender (chi-square test p = 0.302), age, or previous pathology history.

After performing logistic regression (chi-square = 65.159, df = 6, p < 0.001, Hosmer Lemeshow p = 0.878), including vascular surgery procedure, surgical time, anesthesia technique, and ASA ≥3, combined general + locoregional anesthesia remained significantly associated with higher odds of IOH (vs. general alone odds ratio (OR) = 3.41, 95% CI = 1.38-8.43, p = 0.008 vs. locoregional alone OR = 6.37, 95% CI = 1.48-27.47) and surgical time >120 minutes was associated with significantly higher odds of IOH (OR = 7.06, 95% CI = 1.83-27.20, p = 0.004), with an increase in OR of 1.27 and 95% CI of 1.06-1.43 for each 60-minute increment (p < 0.001), with vascular surgery (p = 0.115) and ASA <3 being no longer significantly associated with IOH (p = 0.820).

Postoperative acute kidney injury

Incidence of PO-AKI, excluding patients with previous CKD under dialysis and those with previous AKI, was 14.9% (27 patients, out of 181, developed PO-AKI) (95% CI = 10.3-20.6%), not being related to IOH (OR = 2.3, 95% CI = 0.90-5.66, chi-square test, p = 0.09), consecutive nor cumulative IOH time, neither anesthesia technique (Table 2). PO-AKI was related to surgical specialty (Fisher’s exact test, p = 0.006), with higher incidence after urologic procedures (37.5%, 95% CI = 20.4-57.4%), followed by vascular procedures (16.1%, 95% CI = 8.3-27.3%) and general surgery (9.0%, 95% CI = 4.5-15.8%), and patients with a previous history of pulmonary disease (chi-square test, p=0.023) and CKD (chi-square test, p = 0.044) (Table 2). Forty-eight-hour postoperative creatinine was not evaluated in 45 (22.8%) patients.

**Table 2:** Demographic characteristics of AKI and non-AKI in participants who experienced intraoperative hypotension events. ^a^: After excluding patients with a previous history of chronic kidney disease under dialysis and patients with a previous history of AKI. IQR of 25th to 75th percentile. The Mann-Whitney U test was applied for continuous and ordinal variables and the chi-square test or Fisher’s exact test^b^ was applied for categorical variables. ACE = angiotensin-converting enzyme; ARA = angiotensin receptor antagonist; AKI = acute kidney injury; ASA = American Society of Anesthesiologists; BNP = brain natriuretic peptide; IQR = interquartile range; Hs-TnI = high-sensitivity troponin I; MAC = monitored anesthesia care; MAP = mean arterial pressure

	Participants, N = 181 (91.9%)^a^	Non-AKI participants, N = 154 (85.1%)^a^	AKI participants, N = 27 (14.9%)^a^	P-value
Hypotension (MAP ≤65 for 15 cumulative minutes)	106 (58.6)	86 (55.8)	20 (74.1)	0.092
Cumulative minutes of hypotension: median (IQR)	22 (0–71)	22 (0–74)	44 (6–71)	0.207
Maximum total consecutive number of minutes with MAP ≤65: median (IQR)	0 (0–31)	0 (0–31)	0 (0–37)	0.343
Number of 15-minute consecutive periods with MAP ≤65: median (minimum-maximum)	0 (0–8)	0 (0–8)	0 (0–6)	0.283
Cumulative procedure time with MAP ≤65 (%): median (IQR)	6.38 (0–19.85)	6.02 (0–18.97)	8.03 (0–23.09)	0.432
Administration of intermittent vasopressor	83 (45.9)	68 (44.2)	15 (55.6)	0.301
Postoperative – 24 hours: median (IQR)				
Creatinine, mg/dL	0.74 (0.55–0.95)	0.71 (0.53–0.83)	1.36 (1.08–2.17)	<0.001
Missing	20 (11.0)	19 (12.3)	1 (0.55)	
Postoperative – 48 hours: median (IQR)				
Creatinine, mg/dL	0.65 (0.54–0.92)	0.61 (0.52–0.81)	1.10 (0.84–1.62)	<0.001
Missing	40 (22.1)	39 (25.3)	1 (0.04)	
Other outcomes				
Hospital stay after surgery (days), median (IQR)	9 (5–17)	8 (5–16)	15 (9–28)	0.008
30-day mortality	8 (4.4)	4 (2.6)	4 (14.8)	0.017
Age, median (IQR), years	62 (55–70)	61.5 (54–70)	68 (57–73)	0.073
Female	66 (36.5)	60 (39.0)	6 (22.2)	0.129
ASA class				
I	3 (1.9)	3 (2.2)	0 (0)	0.446
II	60 (37.3)	52 (38.5)	8 (30.8)	
III	85 (52.8)	68 (50.4)	17 (65.4)	
IV	13 (8.1)	12 (8.9)	1 (3.8)	
Missing	20 (11.1)	19 (12.3)	1 (3.7)	
Surgical specialty^b^				
General surgery	100 (55.3)	91 (59.1)	9 (33.3)	0.006
Orthopedics	1 (0.6)	1 (6.5)	0 (0)	
Urology	24 (13.3)	15 (9.74)	9 (33.3)	
Vascular surgery	56 (30.9)	47 (30.5)	9 (33.3)	
Previous medical history				
Cardiovascular disease	96 (53.0)	80 (51.9)	16 (59.3)	0.187
Previous myocardial infarct^b^	13 (7.2)	9 (5.8)	4 (14.8)	0.108
Chronic renal disease (not under dialyses)	10 (5.5)	6 (3.9)	4 (14.8)	0.044
Pulmonary disease	37 (20.4)	27 (17.5)	10 (37.0)	0.035
Diabetes	39 (21.5)	31 (20.1)	8 (29.6)	0.31
Insulin therapy	20 (11.0)	17 (11.0)	3 (11.1)	0.999
Cardiac medication history	181 (100)	154 (100)	27 (100)	
ACE/ARA inhibitor	76 (42.0)	61 (39.6)	15 (55.6)	0.141
Beta-blocker	22 (12.2)	19 (12.3)	3 (11.1)	0.999
Diuretic	19 (10.5)	15 (9.7)	4 (14.8)	0.493
Preoperative: median (IQR)				
Hs-TnI, ng/L	6.3 (2.3–13.35)	6.5 (2.1–14.9)	3.3 (3.1–6.5)	0.646
Missing	133 (73.5)	133 (86.3)	2 (7.4)	
BNP, pg/mL	58.5 (19.4–99.2)	58.5 (20.3–92.4)	56.3 (10.7–217.6)	0.786
Missing	144 (79.6)	125 (81.2)	19 (70.4)	
Creatinine, mg/dL	0.75 (0.62–0.92)	0.74 (0.61–0.86)	0.86 (0.73–1.26)	0.001
Missing	0	0	0	
Intraoperative				
Surgical time, median (IQR), minutes	303 (187–453)	291 (188–430)	334 (167–655)	0.106
Missing	8 (4.4)	6 (3.31)	2 (1.10)	
Anesthesia technique				
General anesthesia	73 (40.6)	63 (41.2)	10 (37.0)	0.689
Locoregional	33 (18.3)	29 (19.0)	4 (14.8)	
Combined	71 (39.4)	58 (37.9)	13 (48.1)	
MAC/Sedation	3 (1.7)	3 (2.0)	0 (0)	
Missing	1 (0.6)	1 (0.6)	0 (0)	

After performing logistic regression (chi-square = 18.021, df = 5, p = 0.003, Hosmer Lemeshow p = 0.361), including all variables with p < 0.1 in univariate analysis (Table 2 - previous history of pulmonary disease, previous CKD, urology surgery procedure, IOH, and age), a previous history of pulmonary disease (OR = 2.84, 95% CI = 1.06-7.62, p = 0.038) and urology surgery procedure remained associated with significantly higher odds of PO-AKI (OR = 3.88, 95% CI = 1.33-11.28, p = 0.013), with IOH (p = 0.244), age (p = 0.379), and CKD (p = 0.376) having no association with PO-AKI after adjustment with the model.

Postoperative mortality and hospital stay

We did not find any association between IOH and 30-day postoperative mortality (seven patients died in the non-IOH group and six in the IOH group, chi-square test, p = 0.565) nor postoperative hospital stay (Mann-Whitney U test, p = 0.711). Development of AKI was significantly associated with higher 30-day postoperative mortality (four (2.6%) patients without PO-AKI vs. four (14.8%) patients with PO-AKI died in the 30 days postoperatively, Fisher’s exact test, p = 0.018, OR = 6.52, 95% CI = 1.52-27.91) and longer hospital stay (median difference of five days, 95% CI = 1-10 days, Mann-Whitnet U test, p = 0.009). After performing logistic regression, excluding patients with a previous history of dialysis and preoperative AKI, (chi-square = 14.397, df = 4, p = 0.006, Hosmer Lemeshow p = 0.969) including all variables with p < 0.1 in univariate analysis (ASA ≤3 (p = 0.043), urologic surgery procedures (p = 0.074), previous pulmonary disease (p = 0.056), PO-AKI (p = 0.018)), the adjusted p-value for the relationship between PO-AKI and mortality was 0.105 (OR = 4.75, 95% CI = 0.72-31.19.

## Discussion

The literature includes over a hundred definitions of hypotension, all employing slightly distinct cut-off values, hindering research into IOH [2,3]. Depending on the IOH definition employed, estimated incidences can range from 5% to 99% [3,5], underscoring the relevance of having a universally established definition of hypotension.

MAP is widely accepted as a better indicator of organ perfusion than systolic blood pressure (SBP) [16]. Unlike SBP, which mainly represents the heart’s generated pressure during systole, MAP includes diastolic pressure, providing for a more comprehensive assessment of vital organ perfusion [17]. This distinction is especially important in surgical settings, where maintaining adequate organ perfusion is key to ensuring patient safety and good outcomes after surgery. Furthermore, MAP’s independence from measuring site and technique, whether using invasive or non-invasive methods, as well as its resistance to damping in measurements contribute to its accuracy. In addition, MAP plays a major role in determining tissue blood flow via autoregulation, emphasizing its importance in perioperative monitoring and management [16].

When defining IOH, the optimal threshold for predicting unfavorable outcomes is MAP ≤65 mmHg maintained over a cumulative duration of 15 minutes [12]. According to several studies, MAP values ≤65 mmHg are linked to unfavorable outcomes such as AKI and mortality in non-cardiac surgical patients [12]. Hypotension can be defined by either single blood pressure values or cumulative time below a threshold, such as MAP ≤65 mmHg. The latter method offers a more comprehensive assessment. This thorough definition considers the duration and severity of hypotensive episodes, providing insights into the cumulative impact on patient outcomes [7]. Research shows that extended exposure to MAP ≤65 mmHg increases the risk of organ damage, pointing out the importance of considering the cumulative time when defining IOH [7,18].

Although absolute thresholds for defining hypotension provide practical benefits in clinical decision-making, relative thresholds have been extensively investigated in research settings. However, comparative analyses of absolute and relative thresholds reveal that employing relative thresholds has no significant advantage, particularly concerning adverse outcomes, such as AKI [17]. Therefore, regardless of preoperative pressure considerations, clinicians can confidently use absolute thresholds to guide intraoperative blood pressure management [18]. Taking these aspects into consideration, we based our IOH definition on the quality indicator from the 2022 Anesthesia Quality Institute (measure ID IIM025) [12].

Our study observed a significant incidence of IOH, affecting more than half of the patients studied, totaling 56.3%. Furthermore, patients experiencing IOH had a median cumulative time of hypotension of 22 minutes, indicating a significant duration of hemodynamic instability during surgery. Very likely, we are underestimating the frequency with which patients have IOH as blood pressure is often only measured on an intermittent basis (every three to five minutes). This implies that brief episodes of low blood pressure may go unnoticed [5]. Moreover, the impact of hypotension extends beyond the intraoperative period, as evidenced by research on individuals undergoing major abdominal surgery. Continuous, non-invasive blood pressure monitoring during the first 48 hours post-surgery revealed that 24% of patients experienced episodes of MAP <70 mmHg for at least 30 minutes, while 18% experienced MAP <65 mmHg for at least 15 minutes. Alarmingly, around 50% of these hypotensive episodes were not detected through routine vital sign evaluations. Plenty of these events could have been avoided, for example, with continuous surveillance on hospital wards, giving rise to the concept of failure to rescue [5].

Initially, our findings showed lower odds of IOH in vascular surgery compared to orthopedics, general, and urology surgeries. However, after logistic regression, vascular surgery no longer exhibited a significant association with IOH. Longer procedures >120 minutes and general + locoregional anesthesia were consistently linked to higher odds of IOH, while lower ASA scores correlated with increased IOH incidence.

Variations among surgical specialties may stem from preferred approaches, such as laparoscopy or laparotomy. The indications of laparoscopy continue to expand and it is increasingly preferred over open surgery, becoming the standard of treatment for the majority of urologic and general surgeries [19,20]. Increased intra-abdominal pressure during laparoscopic surgery may result in reduced ventilation, hypercapnia, a rise in sympathetic nervous system activity, and elevated blood pressure, thereby decreasing the chances of IOH events [21]. These effects might be magnified and may result in greater repercussions in those with preexisting cardiovascular conditions, such as arterial hypertension, characterized by increased baseline blood pressure. As a result, it is possible that our implemented definition of IOH may not adequately account for these circumstances, potentially explaining why lower odds of experiencing IOH were first observed in vascular surgery.

Although previous studies have associated higher ASA scores with IOH, other risk factors not addressed by ASA may explain the observed association between lower ASA scores and increased IOH events in our study [11]. Apart from the factors outlined by the ASA, additional variables such as anesthetic agents may trigger IOH.

Related to anesthesia technique, vasodilation and excessive depth of anesthesia are the primary causes of hypotension during surgery. While spinal and epidural anesthesia cause vasodilatation due to sympathetic block, general anesthesia frequently leads to moderate hypotension due to the effects of intravenous induction and inhalational agents in reducing cardiac output and systemic vascular resistance. Through a synergy of both mechanisms, patients undergoing combined regional and general anesthesia are particularly susceptible to hypotension, corroborating our findings [22].

Therefore, considering procedural characteristics, inclusive type of anesthesia, and duration of the procedure is vital when forecasting and managing IOH. Likewise, preexisting conditions and overall well-being have a significant impact on intraoperative hemodynamics.

Consistent with previous findings in patients undergoing non-cardiac surgery [8,9], our study found a 14.9% incidence of AKI. While previous research has found a significant association between IOH and PO-AKI [6,23], this study found no statistically significant correlation between IOH and the development of PO-AKI. Rather, aspects such as preexisting pulmonary disease and urologic surgical procedures arose as independent predictors of PO-AKI, indicating that a variety of underlying variables contribute to kidney dysfunction after surgery. Several factors have already been linked with an increased risk for perioperative AKI. For both cardiac and non-cardiac surgery populations, preexisting perioperative rise in creatinine (more than 1.2 mg/dL) is a significant indicator for PO-AKI [24-26]. Advanced age, African American race, preexisting hypertension, active congestive heart failure, CKD, pulmonary disease, insulin-dependent diabetes mellitus, peripheral vascular disease, presence of ascites, and high body mass index are independent risk factors for perioperative AKI. A further contributory factor presented is the surgical type, with cardiac surgery linked to the greatest risk for PO-AKI, proceeded by general thoracic, orthopedic, vascular, urologic, and ear, nose, and throat [27,28]. Effect modifiers were defined a priori based on clinical and operative factors that might affect the odds of AKI, such as surgical complexity, fluid management strategies, and anesthetic agents. Nevertheless, considering the lack of accuracy of registries regarding hypertensive drug perfusion and fluid management strategies, we were not able to study the impact of these factors on the relationship between IOH and AKI.

The lack of a clear link in our study could be attributed to a variety of factors, including differences in surgical techniques, patient features, and the number of patients studied. Despite efforts to standardize the definition and detection of IOH and PO-AKI based on established criteria, discrepancies in blood pressure monitoring techniques and thresholds for intervention may affect the accuracy and comparability of our results [29]. Moreover, while all patients had a preoperative creatinine assessment, there was no systematic strategy for postoperative serial creatinine monitoring for all patients, considering all patients underwent high-risk surgery. Due to the unfortunate lack of complete data for the seven-day postoperative period, we focused on the available metrics. We acknowledge that not applying full KDIGO criteria for AKI, such as a rise in serum creatinine by 1.5 times the baseline within a seven-day period and considerations of urinary output, may result in some missed AKI episodes, emphasizing the importance of enhanced renal monitoring. To address this issue thoroughly, future research should prioritize ensuring complete data collection over the entire seven-day postoperative period.

Running counter to previous findings [23], we interestingly failed to discover a statistically significant connection between postoperative mortality and IOH. Nevertheless, additional research is needed to unravel the underlying mechanisms of this complex interaction. Though our study was unable to establish a clear link between IOH and postoperative mortality, it is still critical to thoroughly evaluate the influence of IOH on patient outcomes, particularly in the setting of high-risk non-cardiac procedures.

In univariate analysis, PO-AKI was significantly associated with 30-day postoperative mortality and longer hospital stays. However, after adjusting for other predictors, it lost statistical significance. This implies that whereas PO-AKI may contribute to postoperative morbidity, several factors other than renal function alone may influence its impact on overall postoperative outcomes, including mortality. Early diagnosis of PO-AKI is vital, necessitating active screening in patients undergoing high-risk procedures to intervene promptly and identify those at risk of severe complications, including 30-day postoperative mortality. Implementing new screening strategies for PO-AKI could facilitate early detection, incentivizing the documentation of clinical factors such as postoperative diuresis. Intensive research aims to identify early, specific biomarkers for future clinical use. Therapeutic advances target novel features of AKI pathophysiology, such as inflammatory and oxidative stress responses, endothelial dysfunction, microRNA modulators, and renal-specific vasodilators. Risk stratification systems paired with real-time analytics could assess perioperative AKI risk. Integrating clinical parameters, biomarkers, and electronic medical record data could enable early detection and implementation of renal-protective measures. Early detection is critical as it increases the opportunity window for intervention and prevents subclinical disease from escalating to AKI [29].

Considering our study’s retrospective design and reliance on preexisting medical records, it is crucial to acknowledge inherent limitations. Potential selection bias and inaccuracies due to incomplete documentation may compromise validity. Missing data and the inability to account for all variables further challenge the reliability of the findings. Additionally, the absence of random patient assignment complicates establishing a direct link between IOH and PO-AKI. Generalizing our study is limited as it was conducted in a single academic hospital in Porto, Portugal, with differences in surgical methods, patient demographics, and healthcare infrastructure across institutions. Additionally, the small sample size and homogeneity of patient features weaken the generalizability of our findings. Future research should involve larger and more diverse cohorts to better understand perioperative care dynamics and their effects on renal outcomes, especially in high-risk surgical populations.

## Conclusions

The difficulty in defining IOH has made it challenging to study this complication, leading to varying estimates of how prevalent it is. Our study explores the intricate connections between IOH, PO-AKI, and 30-day postoperative mortality for patients at high risk of complications undergoing non-cardiac elective surgery. Affecting more than half of our patient cohort, our findings highlight how common IOH is and the need for continuous monitoring, individualized care, and considering factors such as anesthesia type and surgery length.

While earlier studies have linked IOH to AKI, our research found no statistically significant association, emphasizing the complex nature of postoperative outcomes. While in initial analyses PO-AKI was significantly related to increased 30-day mortality and longer hospital stays, these associations lost significance after accounting for confounders, reflecting that parameters other than renal function alone influence postoperative outcomes, including mortality. Although our study’s retrospective design and limitations regarding sample size and generalizability restrict our results, our findings underscore the importance of standardizing definitions and monitoring systems for IOH and PO-AKI to enhance early detection and improve patient outcomes, necessitating larger and more diversified research.
